# Heteroscedasticity effects as component to future stock market predictions using RNN-based models

**DOI:** 10.1371/journal.pone.0297641

**Published:** 2024-05-24

**Authors:** Aida Nabilah Sadon, Shuhaida Ismail, Azme Khamis, Muhammad Usman Tariq

**Affiliations:** 1 Universiti Tun Hussein Onn Malaysia, Johor, Malaysia; 2 Abu Dhabi University, Abu Dhabi, United Arab Emirates; UNITEN: Universiti Tenaga Nasional, MALAYSIA

## Abstract

Heteroscedasticity effects are useful for forecasting future stock return volatility. Stock volatility forecasting provides business insight into the stock market, making it valuable information for investors and traders. Predicting stock volatility is a crucial task and challenging. This study proposes a hybrid model that predicts future stock volatility values by considering the heteroscedasticity element of the stock price. The proposed model is a combination of Generalized Autoregressive Conditional Heteroskedasticity (GARCH) and a well-known Recurrent Neural Network (RNN) algorithm Long Short-Term Memory (LSTM). This proposed model is referred to as GARCH-LSTM model. The proposed model is expected to improve prediction accuracy by considering heteroscedasticity elements. First, the GARCH model is employed to estimate the model parameters. After that, the ARCH effect test is used to test the residuals obtained from the model. Any untrained heteroscedasticity element must be found using this step. The hypothesis of the ARCH test yielded a p-value less than 0.05 indicating there is valuable information remaining in the residual, known as heteroscedasticity element. Next, the dataset with heteroscedasticity is then modelled using an LSTM-based RNN algorithm. Experimental results revealed that hybrid GARCH-LSTM had the lowest MAE (7.961), RMSE (10.466), MAPE (0.516) and HMAE (0.005) values compared with a single LSTM. The accuracy of forecasting was also significantly improved by 15% and 13% with hybrid GARCH-LSTM in comparison to single LSTMs. Furthermore, the results reveal that hybrid GARCH-LSTM fully exploits the heteroscedasticity element, which is not captured by the GARCH model estimation, outperforming GARCH models on their own. This finding from this study confirmed that hybrid GARCH-LSTM models are effective forecasting tools for predicting stock price movements. In addition, the proposed model can assist investors in making informed decisions regarding stock prices since it is capable of closely predicting and imitating the observed pattern and trend of KLSE stock prices.

## Ⅰ. Introduction

The stock market is a global network where anyone may purchase stocks, often known as shares of publicly traded companies. Stock, often known as a share or equity, is a form of investment property that reflects ownership in a corporation. In contrast, stock price refers to a stock’s current market value. It reflects the supply and demand for the stock and is influenced by numerous factors, such as the financial performance of the company, macroeconomic factors, international events, political events, social behavior, investor sentiment and etc. [1–3]. Clearly, inflationary pressures and interest rates [4] have a complex political-economic relationship [5].

Stock prices fluctuate constantly and can rise, or fall based on a variety of factors, making them a significant indicator of a company’s financial health. Current stock prices are essential information for investors, especially in terms of the future value of shares [5]. Every investor, from novices to seasoned professionals, seeks the ideal time and decision to trade stocks where an intelligent choice can result in substantial gains and losses for investors [6]. Stock price prediction is an attempt to determine the future value of a company’s stock or stock index. This is an important aspect of investing because it allows investors to make informed decisions regarding the optimal time to buy or sell a specific stock. Predicting stock prices is crucial in the financial and economic world [7].

Predicting changes or movements in stock price has increased investors’ and traders’ interest and demand [8, 9]. Numerous parties, including individual investors, stockbrokers, fund managers, and financial institutions, can benefit from accurate stock price forecasts. An accurate prediction of stock price trends is essential for traders and investors to trade profitably [10]. The profitability of stock market investments has been shown to be substantially correlated with the predictability of stock price changes [11]. Predicting stock price is a difficult task due to various influence factors that affect the stock price [12, 13]. The highly nonlinear structure of stock price time series makes it very difficult to make accurate predictions. As time series are noisy, non-stationary, nonlinearity, and heteroskedastic, predicting volatility for various forms of financial assets [14] is one of the mathematically challenging tasks in time series forecasting.

There are several predictive approaches that have been applied in predicting stock prices and volatility. These approaches consist of either a single method or a hybrid method, such as a combination of mathematical and machine learning techniques. One of the most frequently used method in stock price and volatility forecasting is Box-Jenkins family of models, including Autoregressive Moving Averages (ARMA) and ARIMA models [15, 16]. Given the uncertainty and multitude of factors influencing the stock market, predicting the volatility of stock returns can be more valuable than predicting stock prices directly [14, 17]. Stock return volatility provides vital information about the state and behavior of the stock market, which is of particular interest to investors. Information like this is crucial as it will help investors make the best decisions when trading stocks to maintain profitability in the long term.

Heteroskedasticity, which refers to the uncertainty of variance, is one of the characteristics of stock market volatility. Heteroscedasticity in the volatility series explains the measured volatility transmission of the stock indices [18]. Stock return volatility provides vital information about the state and behavior of the stock market, which is of particular interest to investors [19]. Information like this is crucial as it will help investors make the best decisions when trading stocks in order to maintain profitability in the long term. The Generalized Autoregressive Conditional Heteroskedasticity (GARCH) model, which is part of the Box-Jenkins family, has been widely employed to develop an accurate forecasting model in financial field, particularly in volatility forecasting [20–23]. The variance of the current error term is a function of the variances of prior error terms, as predicted by the GARCH model.

The GARCH model is applied to a case that often exhibits fluctuating conditions, followed by a brief period of relative quiet and could respond to fluctuation sequences better. The model can accurately reflect the regular fluctuations in financial data volatility [24]. Previous study by [25] showed the heteroskedasticity component of stock market returns can be employed to forecast future market value. Factors such as political events [26] and general economic conditions [27] could affect the stock price movements and these factors only can be measures through news and bulletin. Instead of taking risk full step by extracting the qualitative variables, GARCH model can capture the information and news reported within the historical trading days. Despite the ability to capture the volatility in stock price return, GARCH has shown to have limitations in its ability to catch abrupt changes in volatility and an inability to capture non-linear relationships.

With the advancement of soft computing, Deep Learning (DL) algorithm has received massive rise in popularity for forecasting [28]. DL is a learning algorithm derived from neural network. The algorithm comprises of several layers that transform input data to outputs while learning progressively higher-level features. The DL algorithm also comprises a hidden layer. Hidden layers are located in between input and output layer, containing multiple hidden layers. An algorithm with multiple hidden layers is referred to as deep neural network or deep learning. There are two commonly used approaches in DL which are Convolutional Neural Network (CNN) and Recurrent Neural Network (RNN). CNN is a DL algorithm that deals with spatial data such as images, while RNN is suitable for sequential and temporal data [29, 30]. RNN were applied in various area such as in industry of automotive & transportation, healthcare & medicine, retail and more [31].

Given that the stock market is a field that deals with financial time series, DL algorithm based on RNN is more suitable compared to CNN. RNN able to process sequential and temporal data before the advent of attention models. RNN has demonstrated it capability in predicting two different leading stock markets in the world which is National Stock Exchange (NSE) and New York Stock Exchange (NYSE) [15]. When predicting stock return volatility, RNNs have been shown to have some benefits over conventional statistical models such as GARCH.

Long Short-Term Memory (LSTM) and Gated Recurrent Unit (GRU) are the commonly known extensions in RNN. Both LSTM and GRU are highly capable of learning long-term dependencies compared to traditional RNN. LSTM has been demonstrated as an effective and applicable in a range of fields, such as the prediction of stock market value [15], demand forecasting [27], the spread of the COVID-19 virus [32], fake news detection [33], anomalous noise detection [34], entity detection [35], and others. The LSTM has demonstrably proved its supremacy in prediction area. According to a study by [36], the LSTM model outperformed conventional univariate time series prediction approaches. On the other hand, an LSTM prediction model employing up/down reversal point features was developed, and the average prediction accuracy for the Chinese and American stock markets was found to be 68.6% and 55.5%, respectively [37].

Known as modified RNN, GRU has outperformed in many fields and has proven in better performance compared to traditional RNN [38]. GRU demonstrated its superiority in forecasting such as wind power forecasting [39], aquaculture [40], air quality [41] and etc. GRU were also applied in predicting financial time series data. A study conducted on applicability of DL approach on predicting BANKEX data proved that GRU is capable in providing a day-ahead and four-steps ahead of all stock for S&P BSE-BANKEX trends with least error accuracy [10]. Other than that, GRU is also applied in sentiment classification cases since it is able to preserve semantics over time. Previous study by [42] showed that GRU able to able to capture sentimental relationship. However, these RNN algorithms also have limitations such as overfitting, intricacy, and inability to handle long-term dependencies.

In recent years, rather than depending solely on mathematical and traditional statistical methods, researchers have proposed a hybrid model that combines statistical methods and Machine Learning (ML) or DL methods for predicting stock prices. Hybrid models have been found to be effective for dealing with linear and non-linear characteristics in many cases [43] and boost prediction accuracy [44]. Hybrid models are preferable not only in terms of prediction results, but also in terms of robustness and extrapolation capabilities [45]. Hybrid models such as Deep Learning (DL) provide better and clearer results to aid investors’ expectation toward the stock trading. Due to the positive results of previous work, a hybrid of RNN and GARCH may give a better understanding of stock market predictions.

A hybridization model, for example, was demonstrated in [43] to increase forecasting accuracy. As demonstrated by the experimental results of the study, the hybrid ARIMA-ANN model outperformed all three other models used to forecast consumer price indexes (CPI) and the number of cancer patients expected in Yemen’s province. An article by [46] proposed the use of a hybrid LSTM for the Shanghai Stock Exchange (SSE). Among the findings of the study, it was found that the proposed hybrid LSTM model was able to achieve better performance in classifying investor sentiments than the baseline classifiers, and to predict stock prices better than the single model and models without sentiment analysis.

In the past, researchers have suggested that GARCH is ineffective when used as a single forecasting model [47]. Compared to a single model, a hybrid model, preferably with DL algorithm, is expected to overcome the weaknesses of GARCH and to improve forecasting performance [48, 49]. According to recent research on forecasting stock volatility, GARCH combined with LSTM was found to provide the best forecasting model among the other proposed models to predict copper price [47]. The combination of GARCH and LSTM once again demonstrates its credibility in predicting performance with high accuracy while also capturing normal volatility in the stock market [24]. Furthermore, it has been discovered that GRU coupled with GARCH can accurately predict the residual component of long-term satellite degradation under heteroscedasticity [50].

In this study, a hybrid model which combine RNN and GARCH model is proposed to capture the fluctuations and volatility of the Bursa Malaysia stock return and provide a precise forecasting value for trading purposes. This research has two aims: (1) to design a new hybrid model known as GARCH-LSTM, and (2) to evaluate the performance of the proposed model in predicting future stock prices relative to a single LSTM model. It is anticipated that the proposed GARCH-LSTM model will outperform the single LSTM model since the proposed model has an additional heteroskedasticity component. It is expected that the findings of this study will aid investors and economists in making stock trading decisions.

This study offers several contributions: Firstly, it provides step-by-step guidance for selecting the number of input nodes, hidden layers, and output nodes for RNN modeling. Secondly, this study presents a comprehensive overview of combining GARCH with an RNN model by taking the untrained heteroscedastic element from the GARCH model and incorporating it into the RNN model as additional inputs. It is important to note that this is the study’s major contribution. Considering the performance measurements scores obtained in this study in hybrid GARCH-LSTM, it is evident that prediction accuracy has improved.

## Ⅱ. Methodology

### A. Generalized Autoregressive Conditional Heteroscedasticity (GARCH)

The Autoregressive Conditional Heteroscedastic (ARCH) model developed by Engle in 1983 for univariate regression [51, 52]. In 1986, the Generalized Autoregressive Conditional Heteroskedasticity (GARCH) model that uses squared daily log returns to solve conditional volatility problems was introduced. In contrast to the ARCH model, GARCH model considers past conditional variance, also known as volatility. Therefore, volatility remains the same regardless of whether the return is negative or positive [53]. The stock return volatility also known as realized volatility (*RV*_*t*_) on *t* day are computed by using following equation:

$${RV}_{t}=\frac{1}{T}\sum_{j=t}^{j=t+T-1}{{(R}_{j}-\bar{R})}^{2}$$ (1)

Where, *T* is number of trading days after day, *R*_*j*_ is return on day *j*, $\bar{R}$ is an average return on day *j*, and *RV*_*t*_ is actual return volatility. By using closed values from Bursa stock price data, set of daily realized volatility are created and used in training and testing the forecasting models proposed in this study. Due to the nature of financial asset returns and their highly persistent volatility, simple GARCH models turn out to be very effective for modelling and predicting the scale terms [54]. A simple GARCH (*p*, *q*) is explained as follow:

$$\varepsilon_{t}=\eta_{t}\sigma_{t}$$

$$\sigma_{t}^{2}=\alpha_{0}+\alpha_{1}\varepsilon_{t-1}^{2}+\alpha_{2}\varepsilon_{t-2}^{2}+\cdots +\alpha_{q}\varepsilon_{t-q}^{2}$$

$$+\beta_{1}\sigma_{t-1}^{2}+\beta_{2}\sigma_{t-2}^{2}+\cdots +\beta_{p}\sigma_{t-p}^{2}$$ (2)

Where, *ε*_*t*_ is lagged squared residuals, *η*_*t*_ is sequence of independent and identically distributed random variables with zero mean and unit variance, *α*_*q*_ is non-negativity constraints, and *β*_*p*_ is measure the extent to which volatility. As this study is working on heteroscedasticity effect, the ARCH effect test derived by Engle is used to identify ARCH elements in residuals generated by GARCH model estimation. The *H*_0_ for the test indicating there is no ARCH effect found, while *H*_*A*_ indicate there are significant ARCH effects in the dataset.

### B. Long Short-Term Memory (LSTM)

**Fig 1:**
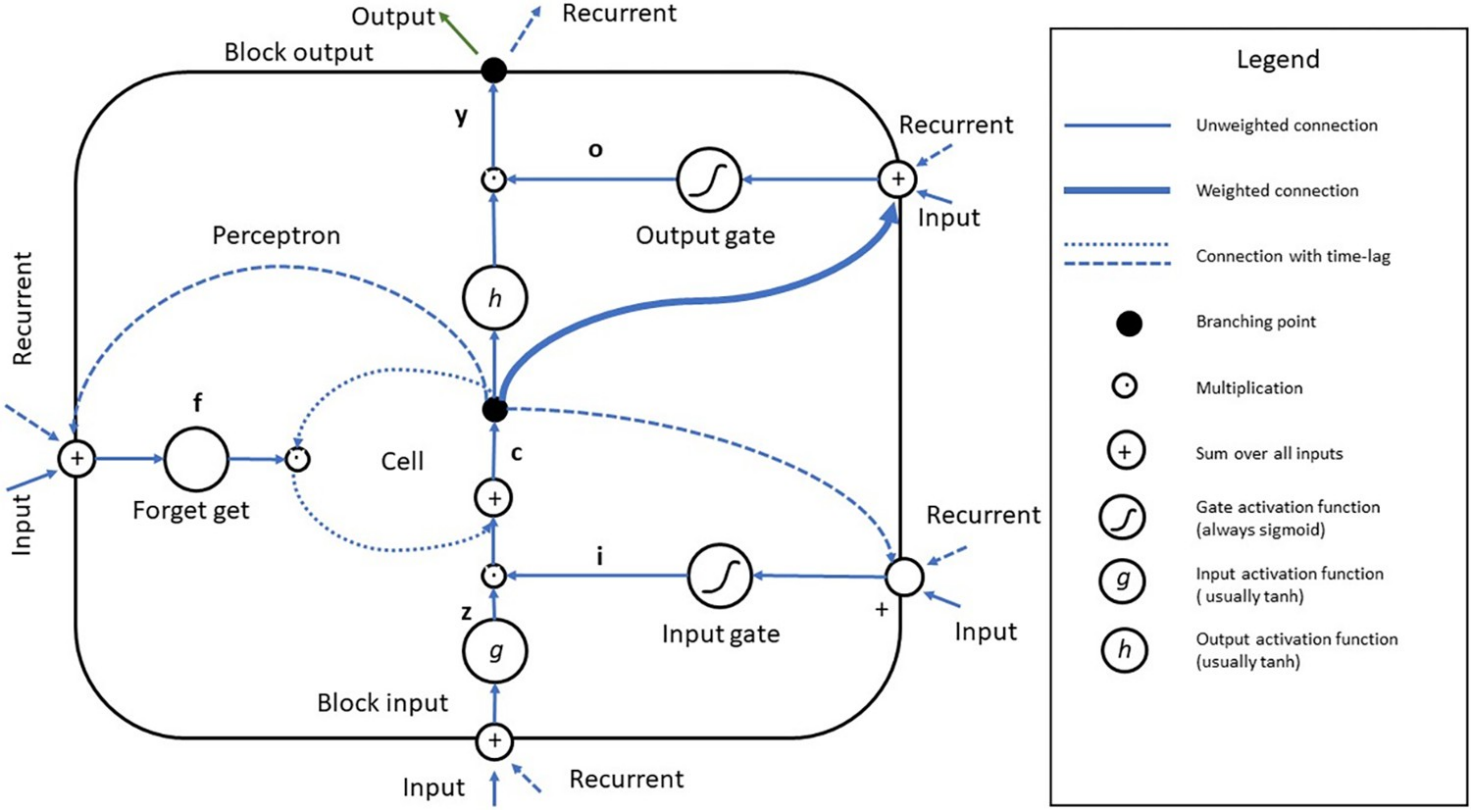
LSTM block diagram.

Fig 1 shows the graphical representation of LSTM algorithm. By using notation from [55] the vector formulas for LSTM layer as follow:

$$Z^{t}=g(W_{Z}X^{t}+R_{Z}y^{t}+b_{Z})$$ (3)

$$i^{t}=\sigma (W_{i}X^{t}+R_{i}y^{t-1}+P_{i}⦿C^{t-1}+b_{i})$$ (4)

$$f^{t}=\sigma (W_{f}X^{t}+R_{f}y^{t-1}+P_{f}⦿C^{t-1}+b_{f})$$ (5)

$$C^{t}=(i^{t}*Z^{t}+f^{t}⦿C^{t-1})$$ (6)

$$O^{t}=\sigma (W_{o}X^{t}+R_{o}y^{t-1}+P_{o}⦿C^{t}+b_{o})$$ (7)

$$y^{t}=O^{t}⦿h(t)$$ (8)

Where, *X*^*t*^ is input vector at time *t*, *W* is rectangular input weight matrices, *R* is square recurrent weight matrices, *P* is peephole weight vectors, *b* is bias vectors and *y* is output at time *t*.

Eq (3) denotes for block input, Eq (4) denotes for input gates, Eq (5) denotes for forget gate, Eq (6) as cell gate, Eq (7) as output gate and Eq (8) denotes as block output. Those gates of sigmoid, tanh, pointwise multiplication, pointwise addition, and vector concatenation can learn and chose which data in a sequence is important to keep or throw away. By doing so, only relevant input will be passed along the chain is sequences to make prediction.

### C. Proposed hybrid model

Stock return volatility is considered key information and helpful for analyzing the movements of the stock market. Predictability of stock market volatility has been noted as a challenging task [56]. Since economists have difficulty in predicting stock market volatility, investors may also find it difficult to analyze their stock markets. This hybrid model is proposed with the aim of helping economists analyze and forecast the future stock volatility values by considering the heteroscedasticity effects contained by stock market that their working with. Previous researchers believed residuals from the GARCH model contained untrained heteroscedasticity effects [56]. To assist investors in analyzing and predicting stock market volatility values more effectively, this study focuses on the development of a hybrid model that incorporates heteroscedasticity effects. Heteroscedasticity element is known as useful information in studying volatility movements.

**Fig 2:**
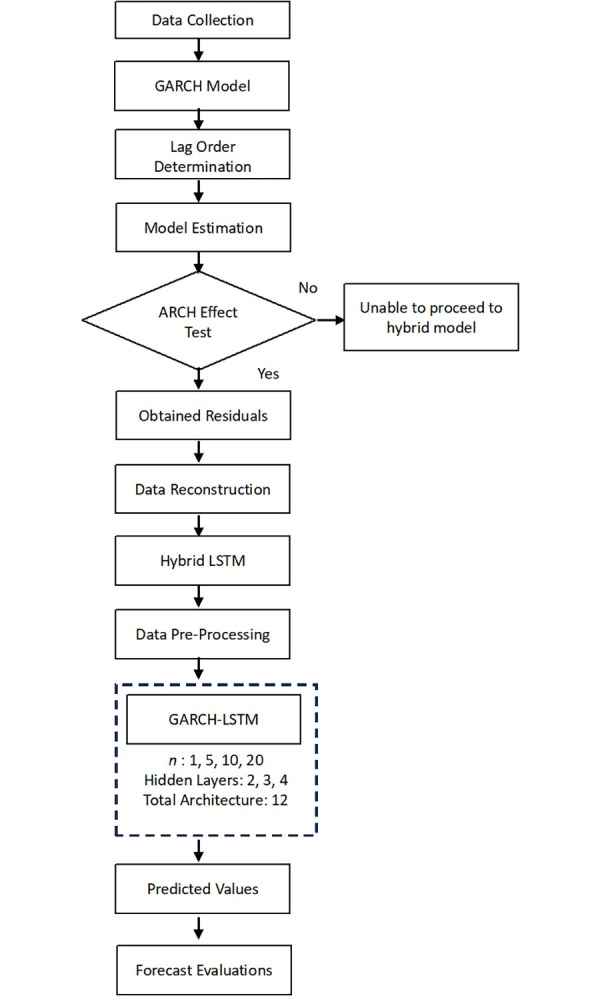
Flowchart of proposed hybrid model.

Fig 2 shows the flowchart of the proposed model. First, the dataset collected from the Yahoo Finance website with time interval in between January 2012 until June 2022 was analyzed. Next, daily return volatility (*RV*_*t*_) was calculated from the dataset. As for the first part, *RV*_*t*_ were trained and tested in GARCH model. Forecasted values produced by GARCH model is then tested using ARCH effect test to determine the heteroscedasticity element left in the residuals produced by the model. Once ARCH effect was detected, residuals from the GARCH model is considered to containing heteroscedasticity element which also meant to be useful information that untrained by the GARCH model. Second part is residuals that was estimated from GARCH model once again used as additional variable to be used in training the daily *RV*_*t*_ in LSTM model. From now on, LSTM are trained and tested using two variables which are *RV*_*t*_ and residuals from the GARCH model.

The hybrid model namely GARCH-LSTM which explain the hybrid of both models and specify three hidden layers of LSTM. Based on the pilot study, the number of hidden layers most optimum in terms of time and performance accuracy is three layers. This hybrid model validated with comparison of single LSTM model that only used dataset of stock price without heteroscedasticity component.

### D. Accuracy measurements

Forecasting accuracy can be measured in a variety of ways. To determine the ability and performance of proposed models in providing accurate predictions, it is imperative that the appropriate accuracy measurements be selected. To evaluate the performance of each model, it is necessary to have at least a measure of absolute error, such as MAE or RMSE [57]. The study employs several performance evaluations, including Root Mean Square Errors (RMSE), Mean Absolute Errors (MAE), Mean Absolute Percentage Errors (MAPE), and Heteroscedasticity Mean Absolute Errors (HMAE).


$$RMSE=\sqrt{\frac{({{RV}_{t}-{\hat{v}}_{t})}^{2}}{n}}$$ (9)

$$MAE=\frac{\left|{RV}_{t}-{\hat{v}}_{t}\right|}{n}$$ (10)

$$MAPE=\frac{100}{n}\left|\sum \frac{{RV}_{t}-{\hat{v}}_{t}}{{RV}_{t}}\right|$$ (11)

$$HMAE=\frac{1}{n}\sum \left|1-{\hat{v}}_{t}/{RV}_{t}\right|$$ (12)

Where *RV*_*t*_ is the actual value at time *t*, $\hat{v_{t}}$ is forecast value at time *t* and *n* is number of sample observation. The accuracy of the models are measured by these metrics, which are indicative of the model’s performance. In general, lower values indicate that the model has fewer errors and is more accurate in predicting future values. A MAE measures the difference between predicted values and actual values, whereas an RMSE measures square root errors and considers their magnitude. In terms of percentages, MAPE is a metric used to measure the difference between predicted and actual values. As an alternative, HMAE is used to describe the difference between the model’s predictions and the actual targets that correspond to those predictions. HMAE is more accurate if it is closer to 0 than it is otherwise [58–60].

### E. Datasets

In this study, the dataset utilized for analyzing KLSE (Kuala Lumpur Stock Exchange) stock prices spans from January 2012 to December 2022. The dataset was sourced from https://www.investing.com, a reputable financial data platform, and encompasses comprehensive historical stock price records. The chosen time frame enables a thorough examination of stock price trends, volatility, and other relevant financial metrics within the specified period. This aids in a comprehensive understanding of KLSE stock market dynamics over the years. In accordance with open data principles, the dataset used in this study is publicly available and can be freely downloaded. The use of the dataset in this research adheres to the platform’s usage policies, which do not impose any restrictions on data sharing, redistribution, or analysis.

**Fig 3:**
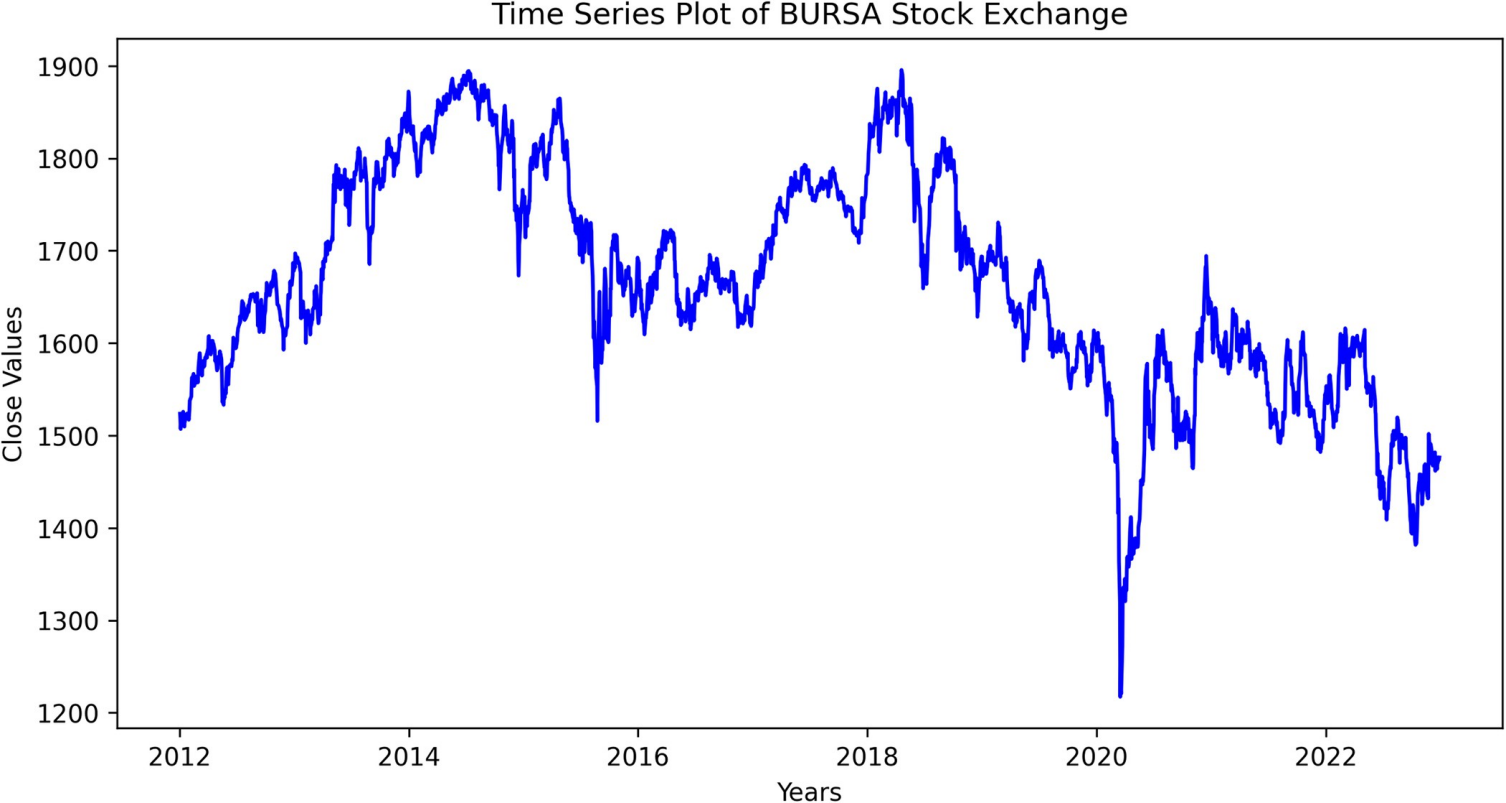
Time series plot of KLSE stock index.

Based on the timeline event in Fig 3, the first COVID19 pandemic outbreak was reported in March 2020. The figure shown KLSE stock index showing a steady growth from 2012 to 2014, then turned downward until mid-2016, and continues to grow every two years. There is a significant downward trend from end-2019 to mid-2020. This is due to the Covid-19 pandemic outbreak and the stock market was impacted in a negative way. As for the seasonal effects, there seem to be cyclical movements across the month of each year, yet it is hard to describe the dataset as random variation. Table 1 showed the excerpt of the dataset used in this study. The dataset is characterized as non-normal and non-stationary by visual inspections. Since financial time series dataset are expected to be this way, it is normal due to its seasonality effects. The dataset must undergo preprocessing step where the average daily return volatility *RV*_*t*_ were calculate. The *RV*_*t*_ were calculated using daily close price of the dataset. The *RV*_*t*_ is then trained and tested in GARCH model for the first part of the analysis.

**Table 1:** Excerpt dataset of daily KLSE stock index.

Date	Open	Close	Returns	*RV*_*t*_	residual (GARCH)
2/2/2012	1526.61	1537.09	0.010332	0.073	-0.00147
3/2/2012	1537.59	1538.77	0.001092	0.073	-0.00152
8/2/2012	1542.27	1553.18	0.009321	0.074	-0.00057
9/2/2012	1555.15	1565.32	0.007786	0.075	0.000409
10/2/2012	1562.72	1561.66	-0.00234	0.076	0.001435
13/2/2012	1560.35	1562.82	0.000742	0.075	0.000413
14/2/2012	1565.25	1566.05	0.002065	0.075	0.000329
15/2/2012	1566.66	1561.3	-0.00304	0.076	0.00182
16/2/2012	1558.27	1550.49	-0.00695	0.081	0.006817

**Fig 4:**
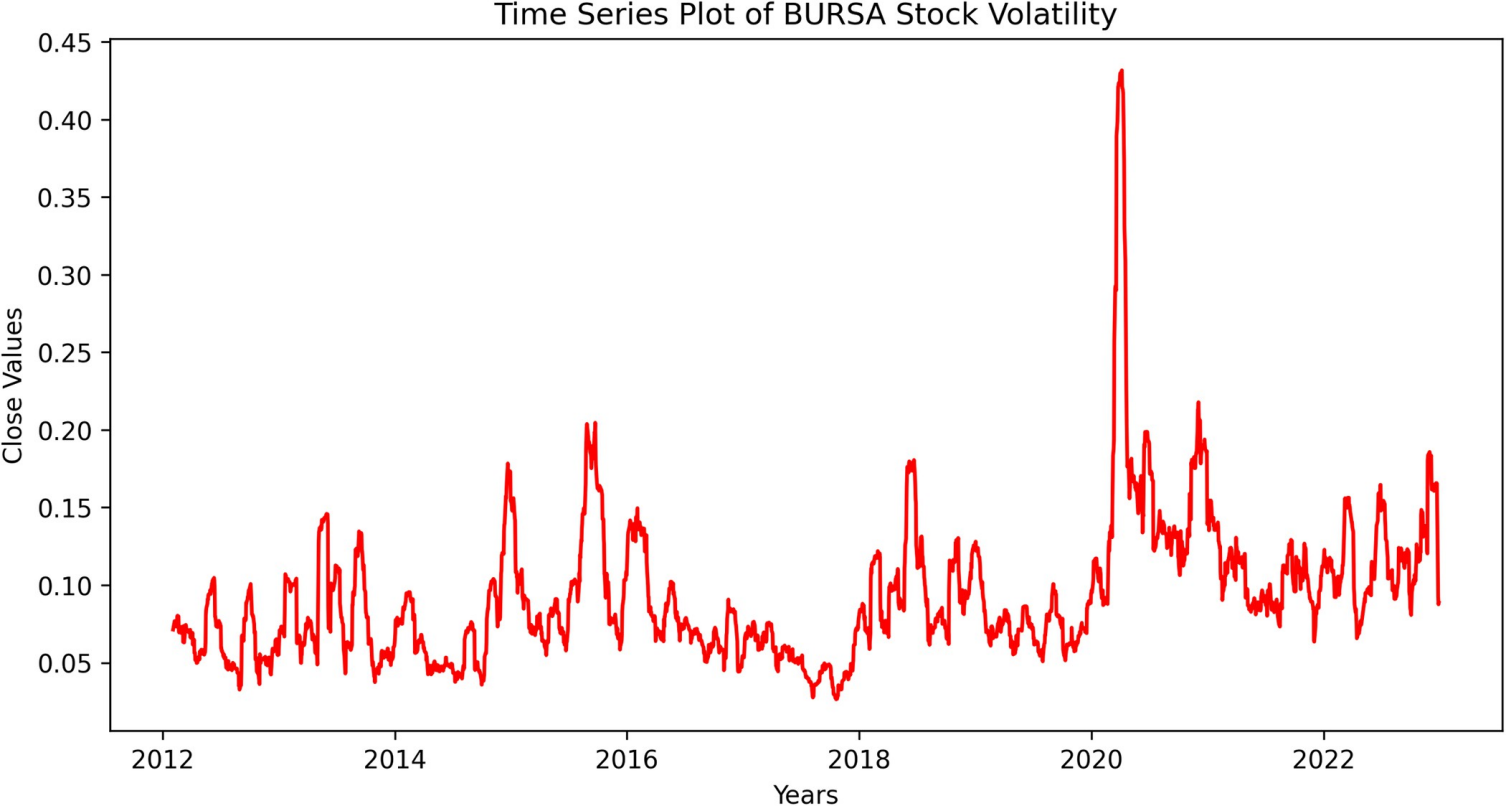
Realized volatility of KLSE stock return.

Fig 4 showed the realized volatility of KLSE Stock Return. Visual inspection revealed that the dataset has high pick volatility especially on early year of 2020 that the dataset has reached the abnormal pick of its usual volatility range.

## Ⅲ Results and discussions

The dataset has to undergo statistical testing to prove the normality and the stationarity of the dataset. The results of preliminary study of Anderson Darling (AD) and Augmented Dickey-Fuller (ADF) tests indicated that the dataset is not normal and not stationary. This characteristic is common for financial time series as these data exhibit substantial fluctuations. AD test resulted in 9.54911 test statistics at 95% confidence interval, indicating the test statistic obtained was larger than critical value of 0.786. Therefore, *H*_0_ is failed to be rejected indicating the dataset is not normal. Meanwhile, the ADF test results showed a *p*-value of -2.5830 and 0.09654 greater than 0.05, which indicates that the *H*_0_ of this test was not rejected. There is no stationary pattern in the sample dataset, indicating that it is non-stationary. Therefore, it does not vary consistently over time and has a time-dependent structure.

### A. GARCH model

**Fig 5:**
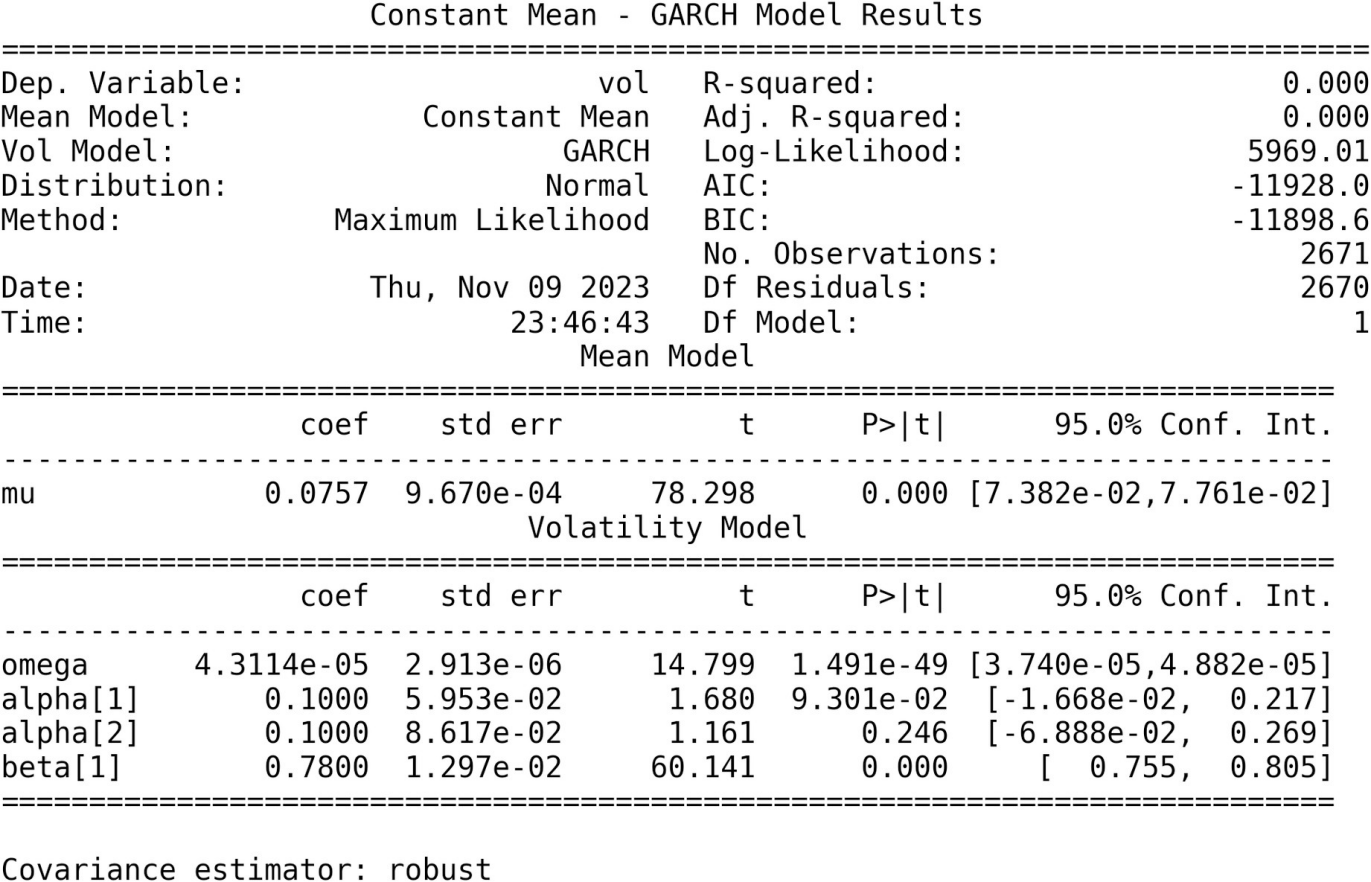
GARCH model estimation.

To get the heteroscedasticity component from the daily stock return, daily stock return is calculated and used in GARCH model as shown in Fig 5. Heteroscedasticity component obtained from residuals of GARCH model. The AIC and BIC scores obtained show this model has good estimation with both scores being -11582.4 and -11559, since the lower the information criteria scores, the better the fit of the model.

The ARCH effect test of ARCH-LM test was conducted on the residuals produced by GARCH model estimation. Based on the *p*-value that was produced by the ARCH-LM tests, if the *p*-value is less than 0.05 at 95% confidence interval, *H*_0_ is rejected. The ARCH effect test was tested on dataset with lag is equal to 10. The full results of ARCH effect test as documented in Table 2.

**Table 2:** ARCH effect test.

Lag	Score	C.V.	P-Value	Present
1	2479.02	3.84	0.0%	TRUE
2	4852.13	5.99	0.0%	TRUE
3	7105.29	7.81	0.0%	TRUE
4	9224.31	9.49	0.0%	TRUE
5	11178.65	11.07	0.0%	TRUE
6	12929.32	12.59	0.0%	TRUE
7	14479.41	14.07	0.0%	TRUE
8	15830.93	15.51	0.0%	TRUE
9	16995.38	16.92	0.0%	TRUE
10	17978.20	18.31	0.0%	TRUE

It can be seen from the above table that ARCH effect has been detected ever since the first lag of the observation was made. As the *p*-values obtained by the tests are below than 0.05, therefore the test failed to accept the *H*_0_ of the hypothesis statement. Therefore, the GARCH model did not fully eliminate the ARCH elements in the dataset. There is still useful information left in the residuals of the GARCH model, which is the uncaptured heteroscedasticity element contained inside the residuals. This condition allows the study to proceed for the hybrid GARCH-LSTM model development.

### B. LSTM model

Since LSTM model has many components to be considered such as number of hidden layers, number of hidden neurons, and number of inputs, this study proposed a simple framework to start developing LSTM architecture that suits the dataset. The architectures of LSTM model with different number of input lag or also called as input node which *n* = 1, 5, 10, 20 and number of hidden layers of 2, 3, 4, 5. Based on pilot study, hidden layer more than five layers required longer time for model execution, yet the performance is equivalent to five hidden layers. It is also proved the model architecture of (20, 3, 1) has reached the highest error forecasting performance. This study used one output node as it is focused on one-day ahead forecasting.

Based on Table 3, the LSTM model with architecture of (10, 2, 1) indicating 10 input nodes, 2 hidden layers and one output node is most sensitive in forecasting KLSE stock index with 12.4815 value of RMSE. To simplify, LSTM (10, 2, 1) taken as the best architecture of LSTM to be used in this study. The same LSTM model chosen also used to be hybridized with GARCH model.

**Table 3:** LSTM architectures.

Input Node	Hidden Layer	MAE	RMSE	MAPE	HMAE
1	2	10.240	13.219	0.658	0.007
	3	9.568	12.600	0.614	0.006
	4	9.764	12.729	0.627	0.006
	5	12.368	15.844	0.795	0.008
5	2	9.800	12.951	0.628	0.006
	3	9.611	12.699	0.616	0.006
	4	9.697	12.815	0.621	0.006
	5	10.576	13.550	0.680	0.007
10	2	**9.354**	**12.041**	**0.599**	**0.006**
	3	9.382	12.174	0.601	0.006
	4	9.892	12.515	0.635	0.006
	5	10.101	13.189	0.649	0.007
20	2	10.295	13.313	0.659	0.007
	3	11.212	14.652	0.721	0.007
	4	10.971	14.145	0.705	0.007
	5	10.987	14.429	0.705	0.007

*Bold indicating the best results

### C. Hybrid GARCH-LSTM model

The hybrid model GARCH-LSTM proposed in this study was designed by incorporating the uncaptured heteroscedasticity element known as residuals from the GARCH model into the LSTM architecture. Even though LSTM with architecture (10, 2, 1) is chosen as the best model, the same list of architectures is used in the hybridization process to find the best hybrid GARCH-LSTM architecture to be used in this study.

Table 4 shows the experimental results for hybrid GARCH-LSTM architecture. The performance results revealed that hybrid GARCH-LSTM architecture of 10-2-1 has the best results with the lowest MAE of 7.961, RMSE of 10.466, MAPE of 0.516 and HMAE of 0.005. As the MAE and RMSE produce lower results, this showed the hybrid GARCH-LSTM models prediction are closer to the actual values, implying higher accuracy. Meanwhile, lower HMAE value indicating the prediction model has less variability. This indicates that the hybrid GARCH-LSTM architecture of 10-2-1, produces the most accurate and reliable prediction compared to other architectures. By combining GARCH for volatility modeling and LSTM for capturing underlying patterns in the data, this hybrid model exhibit higher predictive accuracy compared to standalone models.

**Table 4:** GARCH-LSTM architectures.

Input Node	Hidden Layer	MAE	RMSE	MAPE	HMAE
1	2	8.398	10.805	0.543	0.005
	3	8.633	11.105	0.561	0.006
	4	8.233	10.809	0.533	0.005
	5	20.153	24.798	1.327	0.013
5	2	8.426	10.825	0.546	0.006
	3	8.689	11.324	0.565	0.006
	4	11.543	14.164	0.747	0.008
	5	14.223	17.594	0.920	0.009
10	2	**7.961**	**10.466**	**0.516**	**0.005**
	3	10.038	12.739	0.646	0.007
	4	9.813	12.480	0.638	0.006
	5	14.176	17.638	0.916	0.009
20	2	8.652	11.134	0.560	0.006
	3	9.175	11.859	0.592	0.006
	4	10.589	13.221	0.685	0.007
	5	14.443	17.131	0.943	0.009

*Bold indicating the best results

### D. Comparisons

This study focuses on analyzing Malaysia stock index using both LSTM and GARCH-LSTM. Table 5 illustrated the model’s performance and validated using four statistical performance measurements which are MAE, RMSE, MAPE and HMAE.

**Table 5:** Models comparison.

	LSTM	GARCH-LSTM
MAE	9.354	**7.961**
RMSE	12.041	**10.466**
MAPE	0.599	**0.516**
HMAE	0.006	**0.005**

*Bold indicating the best results

Based on Table 5, both models are used in forecasting stock price index of KLSE and found that model with heteroscedasticity component able to improve the forecasting accuracy. Model performance measurements score obtained by GARCH-LSTM indicated that the model has better forecasting accuracy with 7.961 value of MAE and 10.466 value of RMSE, GARCH-LSTM has improved by 15% and 13% model respectively compared to LSTM model. Furthermore, the ARCH effect test was employed on residual of the hybrid model as shown in Table 6.

**Table 6:** ARCH effect test on GARCH-LSTM model.

Lag	Score	C.V.	P-Value	Present
**1**	0.02	3.84	88.0%	FALSE
**2**	0.50	5.99	77.9%	FALSE
**3**	2.00	7.81	57.3%	FALSE
**4**	2.20	9.49	69.9%	FALSE
**5**	2.21	11.07	81.9%	FALSE

The results from the above table revealed that, statistically evidence all untrained heteroscedasticity element has been used and eliminated during the hybridization process. The test was set to test at five days lags. The test unable to capture the heteroscedasticity element even from the first day lag, resulting in no heteroscedasticity effect left in the residuals. The proposed GARCH-LSTM has fully utilized the heteroscedasticity element, uncaptured by GARCH model estimation, showing it superiority against GARCH model alone.

**Fig 6:**
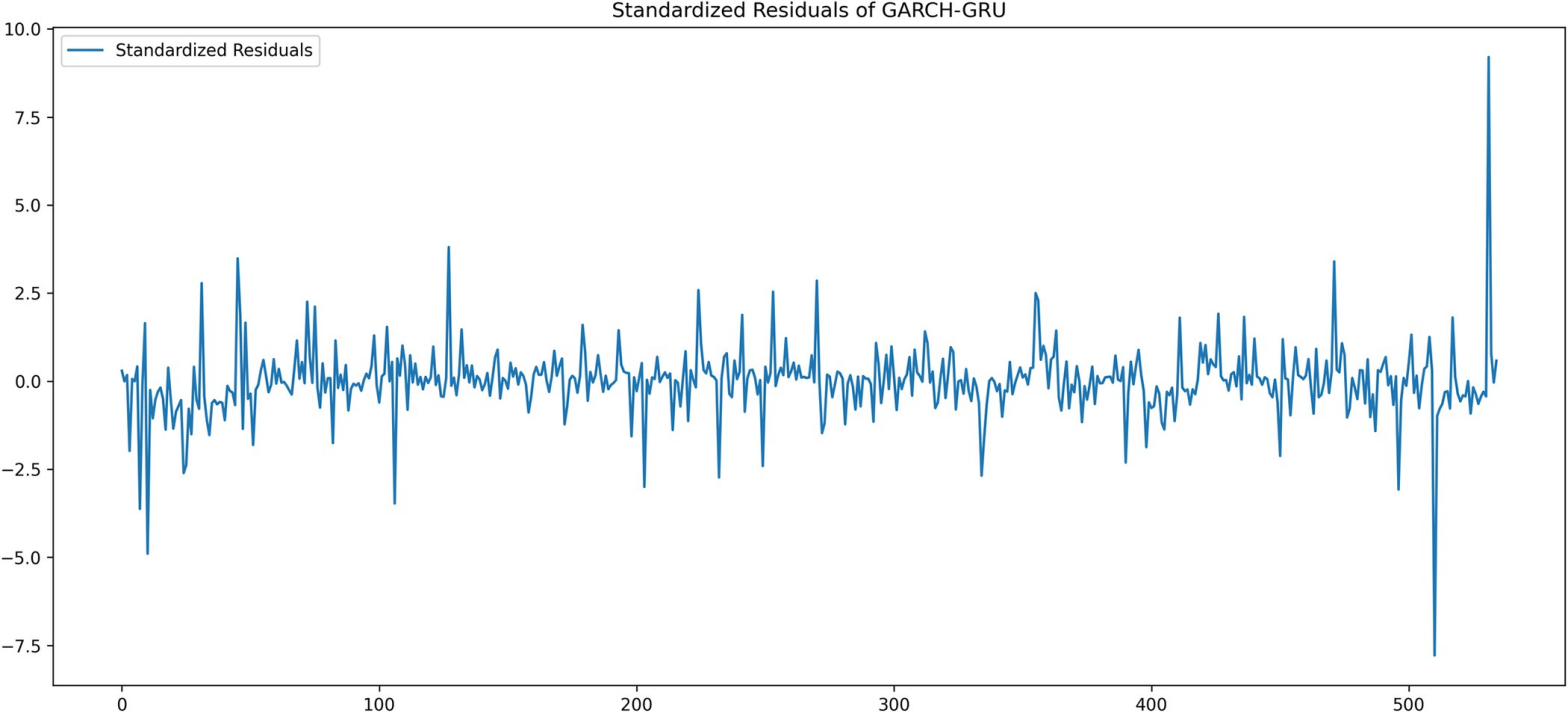
Plot of standardized residuals of GARCH-LSTM.

Fig 6 above shows the plot of standardized residuals of prediction, produced by GARCH-LSTM model in modeling for KLSE Stock index. The plot visually displays a random pattern with most values lying between the zero line as shown in Fig 6. The high points in the plot that were thought to be an outlier, are sparsely and evenly dispersed throughout the zero line. The plot somewhat resembles a random, dispersed white noise pattern that lacks any patterns or clusters.

**Fig 7:**
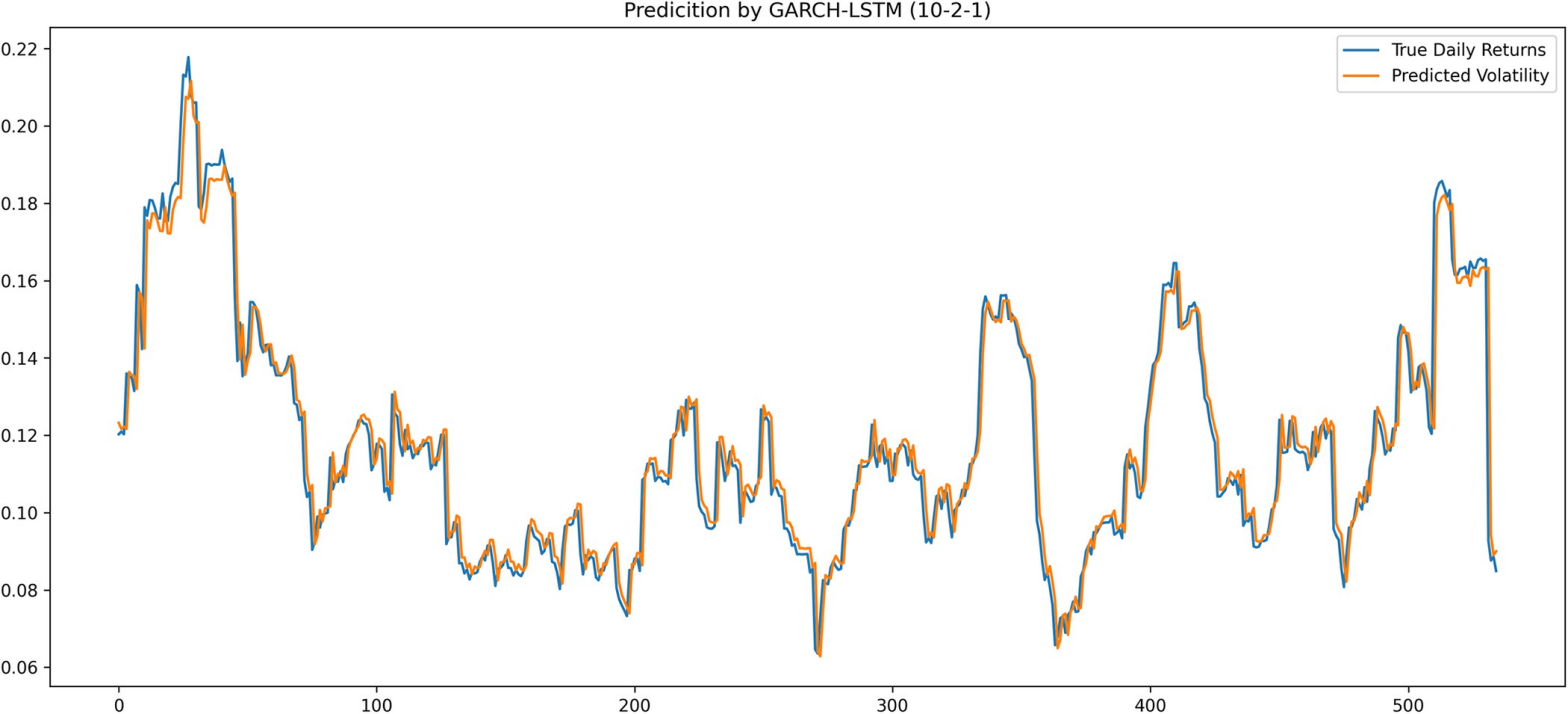
Prediction of Bursa index test set by hybrid model.

Fig 7 illustrated the performance of GARCH-LSTM (10, 2, 1) model and visually analysed that the model is sensitive enough in capturing the stock’s movements even though the dataset has crucial volatility history. The proposed hybrid model has been numerically proven to improve prediction accuracy when compared to the stand-alone LSTM model. As shown in the figure above, GARCH-LSTM capable of imitating the actual value of daily stock price and capturing the index’s ups and downs.

## Ⅳ Conclusion

Since stock market prediction is such an important and difficult task to carry out. There is plenty of ongoing research work for new methods to study these fields, in order to improve the existing approaches and forecasting accuracy. There has been a substantial amount of research on Recurrent Neural Network that has demonstrated how RNN is effective in handling various case studies. This study offers useful insight into the applicability of LSTM, and hybrid GARCH-LSTM in stock price prediction. By adding a heteroscedastic element of the stock market price to LSTM model, instead of simply analyzing single variate information, the forecast accuracy of the model has improved significantly and proven as a reliable model in predicting future value of stock price. According to the findings of this study, hybrid GARCH-LSTM fully utilized the heteroscedasticity element which is often present in financial data and has a difficult time being captured by GARCH models. This suggests that hybrid GARCH-LSTM models are capable predicting future stock price values more accurately than single LSTM models. Results indicated that all objectives set forth earlier were achieved.

Although all objectives were answered and fulfilled, certain limitations were encountered during the study. Finding the GARCH model estimation required a lot of trial and error. The main challenge of this study is to determine the appropriate parameters for the LSTM model. Due to the complexity of RNN and LSTM, finding the appropriate parameters for the LSTM model is difficult since extensive tuning and experimentation are required. The process can be time-consuming and costly. It is also important to note that the right parameters can differ depending on the specific task, making it necessary to tailor them according to each task.

According to this study, several recommendations are made: it is recommended to use a powerful processor in order to conduct the experimental simulations using the RNN-based model. Since this study was conducted using cloud GPU, there are limitations in terms of internet speed and subscription regulation after several usages of the cloud GPU. Furthermore, it is recommended that future research increase the number of inputs, which is the observation of the stock price on a daily basis. Enhanced input datasets enable the model to learn more effectively, ultimately leading to improved forecasting results. A third recommendation is that future research be conducted based on real life demands, such as the number of steps investors require to forecast ahead. Future investigations should prioritize the integration of real-life demand factors to align research with practical applications and market demands. Furthermore, we recommend that future researchers incorporate optimization algorithms when selecting parameters for RNN algorithms and GARCH model estimation. These recommendations emphasize the importance of robust computational resources, enriched input data for the model, and alignment with real-life demand factors to enhance the efficacy and applicability of RNN-based modelling simulations for stock price forecasting. Enhancements are being made to enhance the accuracy and usefulness of predictive models in the financial sector.
